# Plasma-Derived Extracellular Vesicle microRNAs in Multiple Sclerosis: An Exploratory Case-Control Study on Phenotypic Discrimination

**DOI:** 10.3390/ijms27177530

**Published:** 2026-08-22

**Authors:** Oana Vrînceanu, Doina Manu, Smaranda Maier, Claudia Bănescu, Ana-Claudia Carstea, Rodica Bălașa

**Affiliations:** 1 Doctoral School, “George Emil Palade” University of Medicine, Pharmacy, Science, and Technology of Targu Mures, 540142 Targu Mures, Romania; oana.mosora@umfst.ro (O.V.); rodica.balasa@umfst.ro (R.B.); 2 Center for Advanced Medical and Pharmaceutical Research, “George Emil Palade” University of Medicine, Pharmacy, Science, and Technology of Targu Mures, 540142 Targu Mures, Romania; claudia.banescu@umfst.ro (C.B.); clau_genetic@yahoo.com (A.-C.C.); 3 Department of Neurology, “George Emil Palade” University of Medicine, Pharmacy, Science, and Technology of Targu Mures, 540136 Targu Mures, Romania; smaranda.maier@umfst.ro; 4 1st Neurology Clinical, Emergency Clinical County Hospital Targu Mures, 540136 Targu Mures, Romania

**Keywords:** extracellular vesicle miRNA, multiple sclerosis, phenotype, progression

## Abstract

Distinguishing relapsing–remitting multiple sclerosis (RRMS) from secondary-progressive disease (SPMS) remains a clinical challenge, as no single laboratory test reliably identifies the transition. We explored whether plasma extracellular-vesicle (EV) microRNAs could separate the two phenotypes. Four candidate EV-miRNAs: miR-30a-5p, miR-223-5p, miR-155-5p, and miR-146a-5p, were quantified by qRT-PCR in plasma-derived EVs from 13 healthy controls (HC), 7 RRMS, and 16 SPMS patients, normalized to miR-16-5p. EVs were characterized by electron microscopy, dynamic light scattering, and zeta potential, confirming vesicles of expected morphology, size, and negative surface charge. Two miRNAs were informative. miR-155-5p was significantly downregulated in both RRMS and SPMS versus controls but did not differentiate the phenotypes (AUC 0.52), behaving as a disease-general marker. In contrast, miR-223-5p was selectively reduced in SPMS, to approximately one-quarter of control abundance, and provided the only signal distinguishing progressive from relapsing disease (AUC 0.73; rank-biserial −0.46). miR-30a-5p and miR-146a-5p were uninformative. These findings, consistent with the circulating/EV literature, suggest EV-miR-223-5p as a candidate marker of the progressive phenotype and EV-miR-155-5p as a disease indicator. Given the small cohort and near-detection-limit measurements, the study is hypothesis-generating and requires validation in larger, longitudinal cohorts.

## 1. Introduction

Biological markers serve as quantifiable indicators of underlying pathological pathways, offering the distinct advantage of signaling disease progression prior to the manifestation of overt clinical symptoms [1]. When present in biological fluids, including cerebrospinal fluid (CSF), plasma, or serum, these molecules are categorized as soluble biomarkers [2]. Recent technological breakthroughs have revolutionized our capacity to detect these targets [3].

In the context of multiple sclerosis (MS) and related neuroinflammatory disorders, these tools are exceptionally vital. By providing a window into subclinical pathogenic mechanisms, they allow clinicians to intervene early, ultimately mitigating subsequent neurological damage and minimizing long-term disability accumulation [4,5].

The highly variable clinical trajectory of MS and its diverse neurological manifestations continue to present significant diagnostic and management challenges [6]. Relapses serve as the hallmark feature of the relapsing–remitting (RR) phase, characterized as an acute or subacute focal or multifocal central nervous system (CNS) demyelinating event [7]. These episodes must persist for at least 24 h, involve objective clinical findings, and occur in the absence of metabolic stressors such as fever or concurrent infection. While the neurological disturbances associated with relapses are highly heterogeneous, recovery can range from complete resolution to residual, permanent functional loss. Conversely, the secondary progressive (SP) phase typically presents with more uniform motor deficits that drive a steady, irreversible decline in ambulatory capacity [8,9].

Delineating the transition from RRMS to SPMS remains notoriously problematic due to the lack of a standardized, consensus-driven definition for progressive disability accumulation, often complicating phenotypic classification even for experienced clinicians. While the risk of entering the progressive stage scales with overall disease duration, a small subset of individuals remain free of progressive disease for several decades [10]. Furthermore, the underlying biological mechanism driving primary progressive MS (PPMS), wherein 10% to 15% of patients manifest a progressive course from onset without preceding relapsing activity, remains fundamentally unresolved [11]. Crucially, contemporary real-world evidence indicates that the widespread implementation of modern disease-modifying therapies (DMTs) has substantially prolonged the latency period from initial onset to SPMS compared to historical, untreated cohorts [12].

Consequently, identifying an objective, quantifiable biomarker capable of detecting this phenotypic transition at a subclinical stage is of paramount clinical importance, as it would provide an invaluable window for timely, proactive therapeutic optimization before irreversible neurodegeneration ensues [13,14].

From a liquid biopsy perspective, circulating extracellular vesicles (EVs) represent invaluable biomolecular tools for monitoring MS [15]. Structurally defined as minute lipid-membrane spheres, these vesicles orchestrate homeostatic and pathogenic cell-to-cell signaling by transferring functional proteins, lipids, and genomic material (DNA, mRNA, and miRNA) across distant compartments [16,17]. The circulating EV landscape in MS patients varies fundamentally from healthy control cohorts. Clinically, EV dynamics act as sensitive indicators of active neuroinflammation, showing strong correlations with acute relapses and fresh gadolinium-enhancing white matter lesions. Although the field has yet to fully map how distinct MS therapies modulate these vesicular vectors, preliminary studies confirm that treatment regimens can alter blood EV dynamics [18]. Architecturally, the therapeutic and pathogenic importance of EVs relies heavily on their dense miRNA cargo, which not only dictates the downstream molecular cascades of MS pathogenesis but has also been implicated in mediating drug resistance [19].

MicroRNAs (miRNAs) are evolutionary highly conserved non-coding molecules estimated to regulate more than 30% of the human genome, thereby exerting post-transcriptional control over crucial cellular functions, including proliferation, differentiation, cell cycle progression, and apoptosis [20,21]. Aberrant miRNA expression landscapes have been widely documented in the etiology and clinical progression of numerous disorders, particularly neurodegenerative and autoimmune diseases. This extensive regulatory scope, combined with their reproducible dysregulation in neurological conditions, underscores the role of miRNAs as pivotal regulatory nodes within the epigenetic machinery [22]. Because a single miRNA possesses the capacity to simultaneously target multiple messenger RNA (mRNA) transcripts and modulate interconnected biochemical pathways, manipulating these molecules could shift the therapeutic paradigm from basic symptom management to the direct modification of underlying disease trajectories by correcting upstream epigenetic networks [23,24].

**Figure 1:**
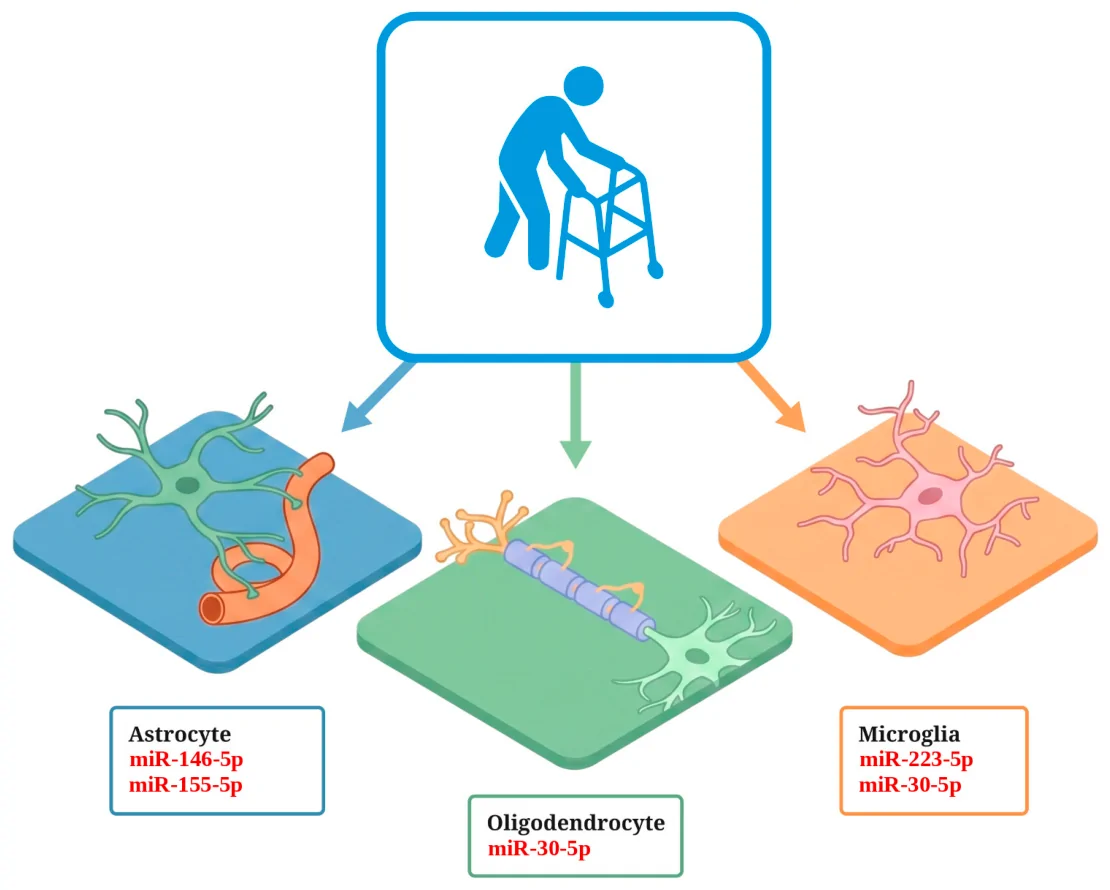
MicroRNA-mediated regulation of glial cells contributing to physical disability worsening and phenotypic progression in MS.

Within the specific context of MS, circulating and tissue-specific miRNAs have emerged as critical drivers of both peripheral immunopathology and CNS tissue remodeling. Specific signatures, such as miR-155-5p and miR-146a-5p, are profoundly involved in orchestrating blood–brain barrier (BBB) disruption, driving pathogenic T-helper (Th1/Th17) cell differentiation, and modulating microglial polarization within demyelinating lesions [25,26]. Concurrently, others like miR-30a-5p, and miR-223-5p act as indispensable regulators of oligodendrocyte precursor cell maturation and central remyelination [27,28]. Given their remarkable structural stability in biofluids like serum and cerebrospinal fluid (CSF), tracking longitudinal changes in MS-specific miRNA profiles offers highly sensitive biomarker potential. This provides a molecular window to forecast clinical relapses, monitor subclinical lesion accumulation, map Progression Independent of Relapse Activity (PIRA), and identify the critical inflection point where relapsing–remitting disease transitions into secondary progression as shown in Figure 1 [29,30].

## 2. Results

### 2.1. Study Design and Methodology Overview

Four candidate extracellular vesicle-associated microRNAs (EV-miRNAs), miR-30a-5p, miR-223-5p, miR-155-5p and miR-146a-5p, were quantified by qRT-PCR in plasma-derived EVs from 13 healthy controls (HC), 7 patients with RRMS and 16 patients with SPMS, with the aim of identifying circulating EV-miRNAs that distinguish the two clinical phenotypes. Expression of each target was normalized to the endogenous reference miR-16-5p using the comparative threshold-cycle (ΔΔCq) method, and relative quantity (RQ, fold change) was calculated as 2^−ΔΔCq^; because ΔCq is inversely related to abundance, a higher ΔCq denotes lower expression and an RQ below 1 denotes downregulation relative to controls. Findings are reported first as a panel-wide overview, then for each individual miRNA, and finally as a direct head-to-head discrimination analysis of the two phenotypes.

**Figure 2:**
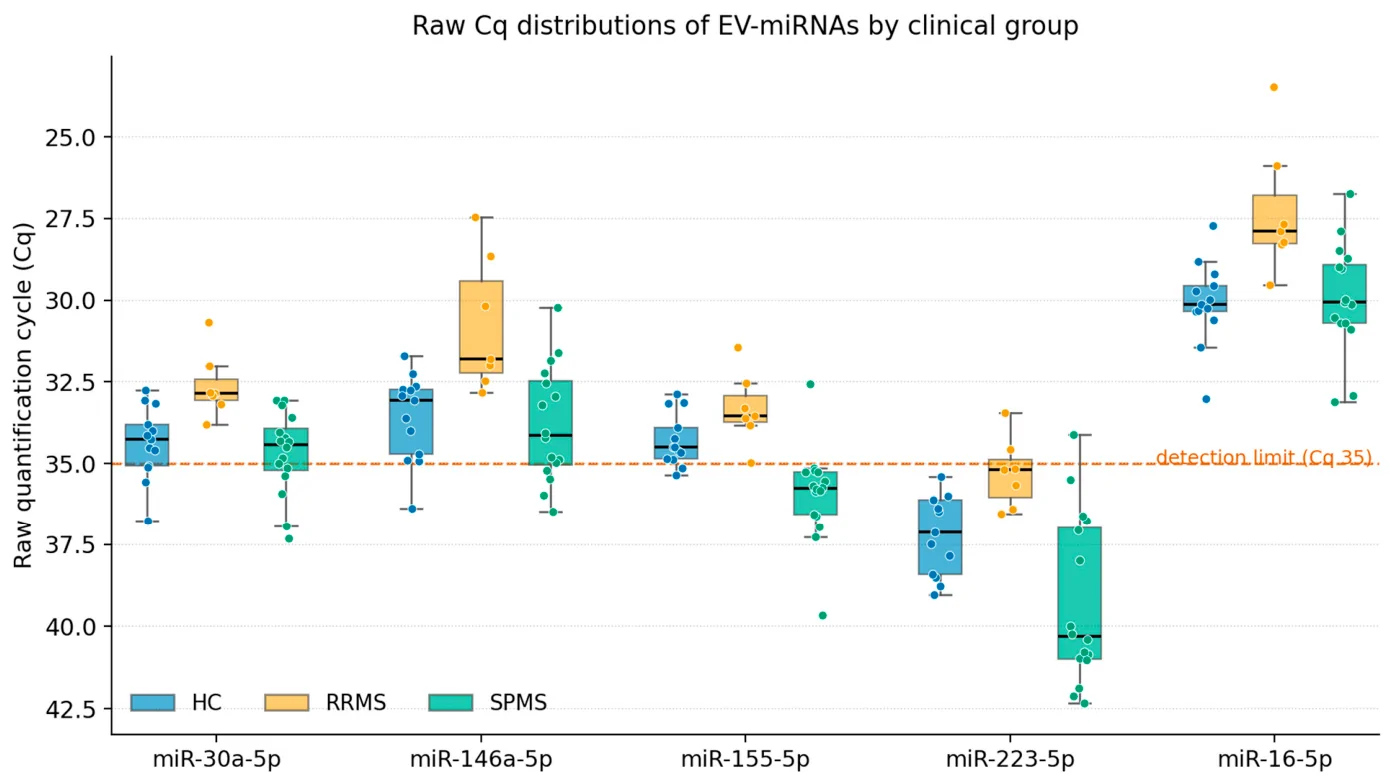
Raw Cq distributions by target and clinical group. Boxes show median and interquartile range with individual samples overlaid; the y-axis inverted so that lower Cq (higher abundance) is at the top. The dashed line marks the Cq 35 reliable-detection threshold. miR-30a-5p, miR-146-5p ant miR-16-5p reference amplify well within the detectable range, whereas miR-223-5p lies at or beyond the threshold in nearly all samples and miR-155-5p crosses it specifically in the SPMS group.

As shown in Table 1 and Figure 2, the reference miR-16-5p and the two robustly expressed targets miR-30a-5p and miR-146-5p amplified within the reliably detectable range in the great majority of samples (median Cq 28–34, detection rates 69–100%), so their measurements, including the analytically informative near-null results for miR-146-5p are trustworthy. In contrast, miR-223-5p was quantified at or beyond the detection limit throughout, its median raw Cq was 37.1 overall, with 33 of 36 samples above Cq 35 (0% detected <Cq 35 in HC, 6% in SPMS) and a median inter-replicate dispersion of 0.71 Cq, indicating consistently late, high-variance amplification. miR-155-5p occupied an intermediate position, being reliably detected in HC and RRMS (85–100% < Cq 35) but crossing the threshold in the SPMS group (median 35.8).

### 2.2. Comparative Panel Analysis

Across the four targets, two miRNAs (miR-223-5p and miR-155-5p) were informative and two (miR-30a-5p and miR-146a-5p) were not. Group-level expression and fold-changes relative to controls are summarized in Table 2, the corresponding inferential tests in Table 3, and the subject-level distributions in Figure 2. Relative to controls, all three remaining targets were reduced in the disease groups, the only exception being miR-146a-5p in RRMS, which was essentially unchanged (FC ≈ 1.01). The largest single change anywhere in the panel was the reduction of miR-223-5p in SPMS to approximately one-quarter of control abundance (FC 0.24).

In the omnibus Kruskal–Wallis test, only miR-223-5p and miR-155-5p differed across the three groups (FDR = 0.024 for both), whereas miR-30a-5p and miR-146a-5p did not (FDR = 0.18). Pairwise comparisons localized these effects (Table 3). miR-155-5p was significantly downregulated in both RRMS (*p* = 0.014, FDR 0.019) and SPMS (*p* = 0.006, FDR 0.012) relative to controls but did not differ between the two phenotypes (*p* = 0.922). In contrast, miR-223-5p was significantly reduced only in SPMS versus HC (*p* = 0.005, FDR 0.021) and provided the sole signal between phenotypes (SPMS vs. RRMS, *p* = 0.089). These results indicate that miR-155-5p tracked the presence of disease, whereas miR-223-5p tracked the progressive phenotype.

**Figure 3:**
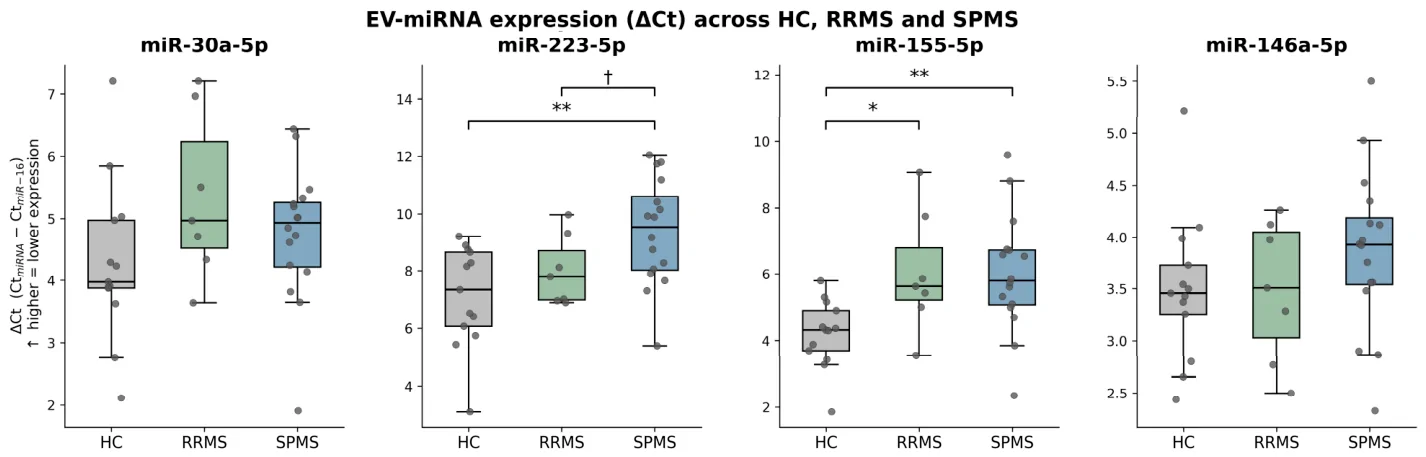
Subject-level EVs ΔCq for each miRNA across HC, RRMS and SPMS. Boxes show median and interquartile range with individual data points overlaid; because the y-axis is ΔCq, boxes positioned higher correspond to lower expression. Significance brackets: † 0.05 ≤ *p* < 0.10, * *p* < 0.05, ** *p* < 0.01 (Mann–Whitney U).

Consistent with the formal tests, the miR-223-5p panel in Figure 3 shows the SPMS distribution shifted toward lower expression relative to both HC and RRMS, while for miR-155-5p both disease groups were shifted by a similar amount and overlapped one another. The miR-30a-5p and miR-146a-5p panels showed broad, overlapping distributions in keeping with their non-significant tests.

### 2.3. Findings by Individual miRNAs

#### 2.3.1. miR-223-5p

**Figure 4:**
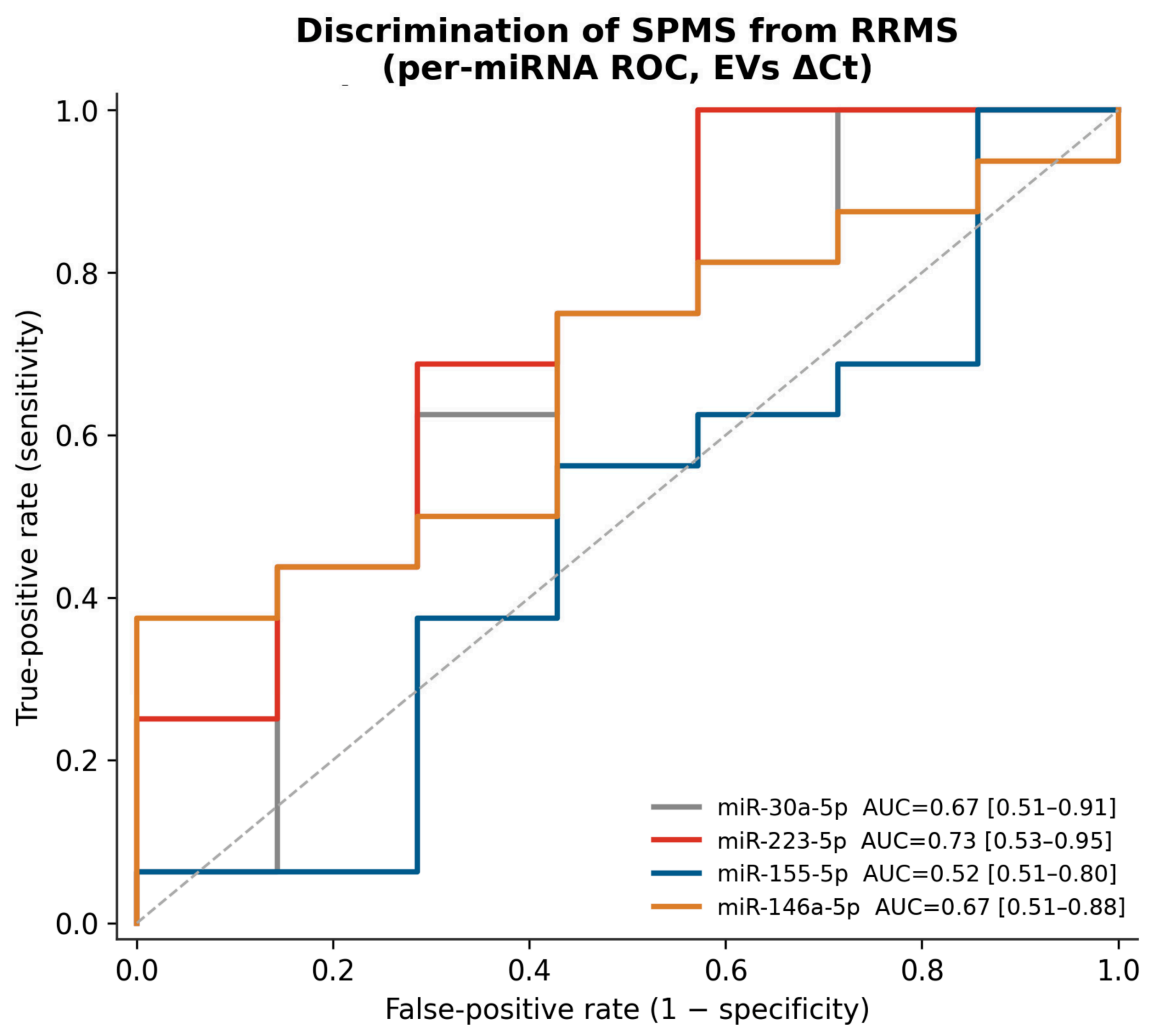
Per-miRNA ROC curves for discriminating SPMS from RRMS (subject-level ΔCq). The diagonal represents chance. miR-223-5p lay highest (AUC 0.73) and miR-155-5p followed the diagonal (AUC 0.52). Curves are step-like with wide confidence intervals owing to the small groups (RRMS n = 7) and should be read as exploratory ranking rather than as a validated classifier.

miR-223-5p was the most strongly dysregulated target and the only one with phenotype-discriminating behaviour. It was reduced to approximately one-quarter of control abundance in SPMS (FC 0.24), the largest effect in the panel, and this reduction remained significant after correction (SPMS vs. HC *p* = 0.005, FDR 0.021; Table 1 and Table 2). In RRMS the reduction was modest and non-significant (FC 0.54, *p* = 0.351), indicating that the loss of miR-223-5p was largely confined to the progressive form. In the direct phenotype comparison, it was approximately 2.3-fold lower in SPMS than in RRMS, carried the largest effect size of any marker (rank-biserial = −0.46) and the best discrimination (AUC 0.73; Table 3, Figure 2 and Figure 4), and was the only miRNA whose phenotype comparison approached significance (*p* = 0.089). Its EVs abundance was very low, so this is the most promising lead in the study but requires confirmation. Given this near-detection-limit quantification, confirmation with a more sensitive absolute method such as droplet digital PCR (ddPCR) will be essential before EV-miR-223-5p can be regarded as a reliable marker of the progressive phenotype.

#### 2.3.2. miR-155-5p

miR-155-5p behaved as a disease-general marker. It was significantly downregulated relative to controls in both phenotypes, to roughly one-third of HC level in RRMS (FC 0.28, FDR 0.019) and SPMS (FC 0.32, FDR 0.012; Table 1 and Table 2), yet the two disease groups were almost identical to one another (FC 1.16, *p* = 0.922) and it did not separate them (AUC 0.52; Table 3, Figure 2, Figure 3 and Figure 4). miR-155-5p was consistently reduced in both disease groups in this cohort, suggesting an association with the presence of MS, but carried no information about clinical phenotype.

#### 2.3.3. miR-30a-5p

miR-30a-5p showed a consistent downward trend in both disease groups versus controls (FC 0.48 in RRMS, 0.74 in SPMS; Table 1) but did not reach significance in the omnibus test (KW FDR 0.18) or in any pairwise comparison (Table 2). Its head-to-head discrimination of the phenotypes was modest and non-significant (AUC 0.67, *p* = 0.222; Table 3). It was therefore not a reliable individual marker in this cohort, although its directionally consistent reduction may warrant reassessment in a larger sample.

#### 2.3.4. miR-146a-5p

miR-146a-5p was the least informative target. It was essentially unchanged in RRMS (FC 1.01) and only mildly, non-significantly reduced in SPMS (FC 0.72, SPMS vs. HC *p* = 0.083; Table 1 and Table 2), and it did not distinguish the phenotypes (*p* = 0.222, AUC 0.67; Table 3). It was, however, among the most robustly detected targets technically, making its near-null result analytically trustworthy.

### 2.4. Identification of Progressive Phenotypes

**Figure 5:**
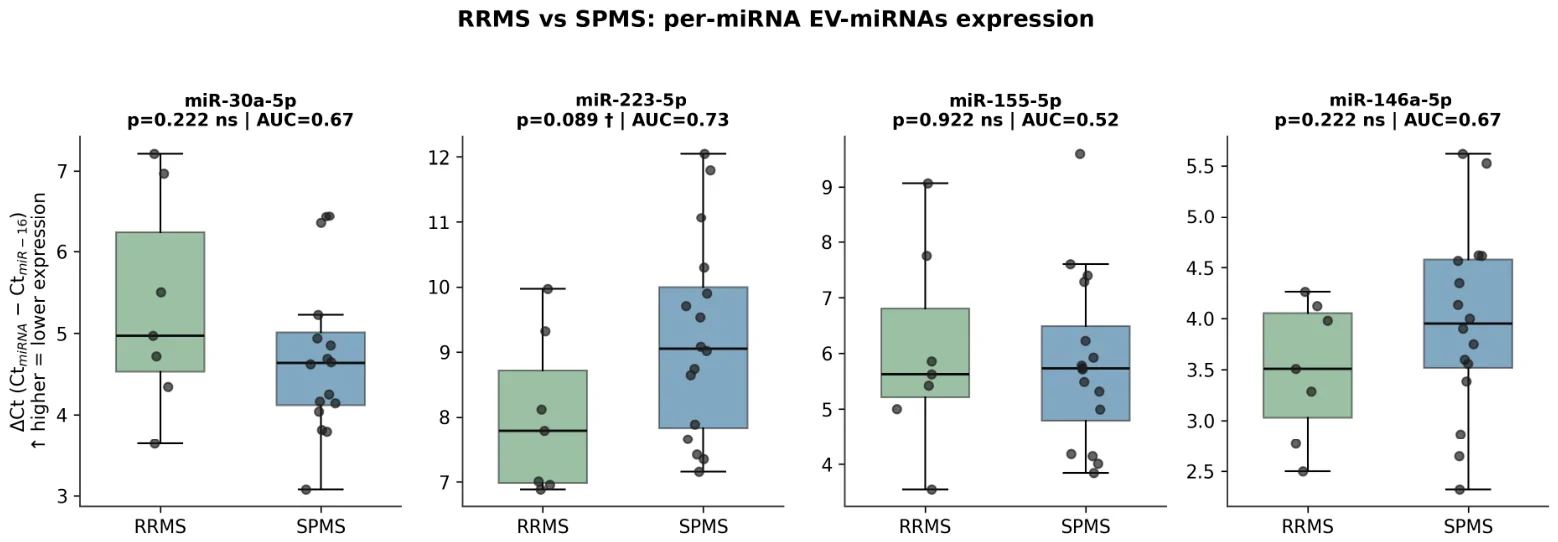
RRMS vs. SPMS EVs expression for each miRNA, with individual data points; each panel is annotated with the Mann–Whitney *p*-value and the SPMS-vs.-RRMS AUC. The miR-223-5p panel shows the SPMS box raised above RRMS (lower expression), the only panel with visible separation; the miR-155-5p panel shows two near-superimposed boxes. Significance markers: † 0.05 ≤ *p* < 0.10 (trend); “ns”, not significant (*p* ≥ 0.10). Points represent individual subjects.

**Figure 6:**
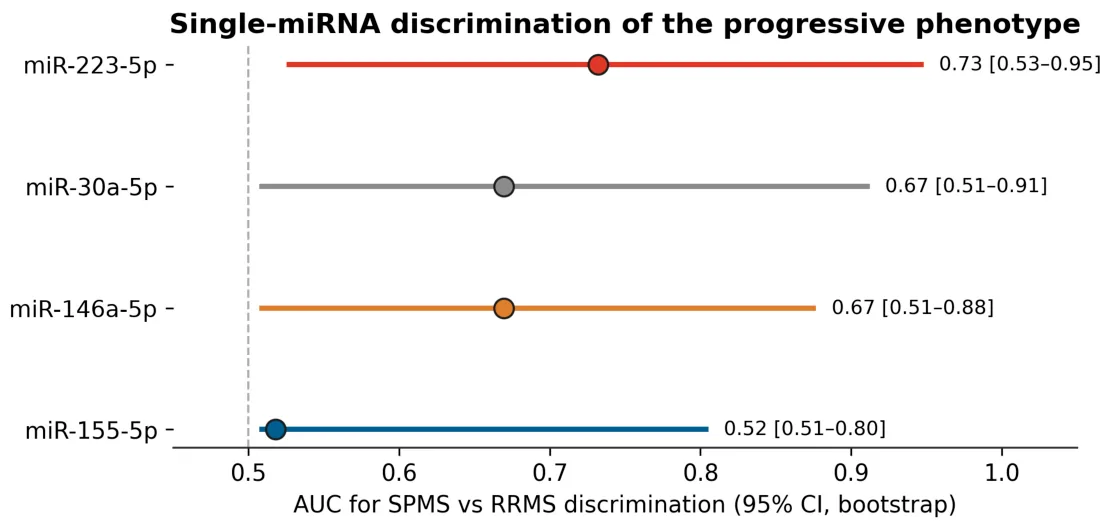
Single-miRNA discrimination of the progressive phenotype, ranked by AUC with bootstrap 95% confidence intervals; the dashed line denotes chance (0.5). Only miR-223-5p had a point estimate clearly above chance, though its confidence interval, like those of the other markers, still extended toward 0.5, quantifying the larger samples needed before the result can be considered firm.

Because the separation of RRMS from SPMS is the central clinical question, the two groups were compared directly using effect-size and receiver-operating-characteristic (ROC) analysis (Table 4, Figure 4, Figure 5 and Figure 6). Only miR-223-5p carried a moderate-to-large, directionally clear separation (SPMS lower; rank-biserial −0.46, AUC 0.73), while miR-155-5p performed at chance (AUC 0.52).

## 3. Discussion

This study profiled EV-miRNAs in plasma-derived EVs from patients with RRMS and SPMS and from HCs, with the specific aim of identifying circulating markers able to separate the two clinical phenotypes. Two findings dominated. miR-223-5p was selectively reduced in SPMS and provided the only signal, across fold change, effect size and ROC analysis, that distinguished the progressive from the relapsing phenotype, whereas miR-155-5p was reduced in both disease groups relative to controls yet did not separate them, behaving as a disease-general rather than a phenotype-specific marker. miR-30a-5p and miR-146a-5p were not informative in this cohort. The distinction between a marker that tracks disease and one that tracks the progressive phenotype is clinically meaningful: there is currently no single laboratory test that identifies the conversion from RRMS to SPMS, which remains a retrospective diagnosis based on the gradual accumulation of disability, and circulating biomarkers that flag this transition are an explicit unmet need [31,32].

We deliberately measured miRNAs within plasma of EVs rather than total cell-free circulating miRNA. The choice of blood compartment is not trivial: serum and plasma yield systemically different EVs and miRNAs profiles, because clot formation and the accompanying platelet activation release additional vesicles and miRNAs into serum, inflating and distorting the measured signal relative to plasma [33,34]. We therefore analysed EDTA-anticoagulated plasma, which more faithfully reflects the circulating vesicle pool and minimises coagulation-related contamination. EVs are secreted by both immune and CNS cells, can cross the BBB, and provide a protected, partly selectively packaged RNA cargo; for these reasons EVs miRNA profiles in MS are distinct from, and have been argued to be at least as informative as, free circulating profiles [32,35]. This compartmental distinction is central to interpreting our results, because much of the existing literature on these four miRNAs derives from cells or tissue, where the direction of change is frequently opposite to that seen in biofluids.

The miR-223-5p literature in MS is directionally heterogeneous and strongly compartment-dependent. In cellular and lesional material, miR-223-5p is consistently reported as upregulated: it is elevated in active demyelinating lesions [36], in peripheral blood mononuclear cells (PBMCs), more markedly in RRMS [37], in CD4^+^ T cells during the relapsing phase [38], and in myeloid-derived suppressor cells, where it regulates their number and suppressive function in both MS and experimental autoimmune encephalomyelitis (EAE) [39]. In the circulating compartment, however, the picture inverts: serum miR-223-5p is decreased in MS, and circulating levels correlate inversely with disability in progressive disease [40], while a four-year longitudinal study found serum miR-223-3p to correlate negatively with T1 lesion volume in SPMS and PPMS [41]. Our EVs data align with this circulating, progression-associated literature rather than with the cellular upregulation, and they are concordant with the observation that EVs miR-223-3p formed part of a multi-miRNA serum-EVs signature separating relapsing from progressive MS [32].

miR-155 is the prototypical inflammatory miRNA in MS. It is upregulated in active lesions, PBMCs and CD14+ monocytes [42,43], is induced in brain endothelium where it disrupts BBB integrity and promotes leukocyte adhesion [26,44], drives Th1/Th17 differentiation and pro-inflammatory myeloid polarization [45,46], and its genetic deletion or silencing renders mice resistant to EAE [47]; together with miR-146a-5p it is induced downstream of NF-κB in MS-affected tissue [48]. On the strength of these roles, it has repeatedly been proposed as a candidate marker of the RRMS-to-SPMS transition [31]. In our EVs dataset, however, miR-155-5p was downregulated in both phenotypes and showed essentially no ability to separate them (AUC 0.52). The downregulation again illustrates the discordance between cellular upregulation and the circulating/EVs compartment, where selective packaging and treatment effects operate and circulating inflamma-miRs, including miR-155-5p are, for instance, reduced by disease-modifying therapy [49]. The key point for biomarker development is that, in this compartment, miR-155-5p reliably flagged the presence of disease but carried no phenotype information, which tempers its proposed value as a transition marker even as it supports its use as a general indicator of MS.

miR-146a-5p is an NF-κB-driven negative-feedback regulator of innate immunity that is upregulated in PBMCs, white-matter lesions and the CSF of patients with active disease [46,48]. Its behaviour in biofluids is inconsistent: circulating plasma miR-146a-5p has been reported as lower in RRMS and associated with disability progression [47], whereas other serum studies describe elevation in progressive forms [49,50]. Our finding, essentially unchanged in RRMS and only mildly, non-significantly reduced in SPMS, sits within this heterogeneity. Importantly, miR-146a-5p was among the most robustly detected targets technically, so its near-null result is analytically trustworthy rather than an artefact of poor detection. For miR-30a-5p, direct MS evidence is sparse, although other members of the miR-30 family (notably miR-30b-5p) recur in EVs RRMS signatures [32] and in erythrocyte miRNA profiles of RRMS [51,52]. The consistent but non-significant downward trend we observed for miR-30a-5p in both disease groups may merit reassessment in a larger sample but does not support its use as an individual marker here.

Despite its exploratory nature, this study addresses a recognised gap in MS care: the absence of a minimally invasive test capable of separating relapsing from progressive disease, a distinction that currently rests on the retrospective clinical accumulation of disability. By interrogating the plasma EVs compartment specifically, rather than total circulating or cellular miRNA, it contributes to a small but growing body of work indicating that EVs-packaged miRNAs carry phenotype-relevant information, and it offers a conceptually useful separation between a disease-general marker (miR-155-5p) and a phenotype-associated one (miR-223-5p) that could help structure future biomarker panels.

These contributions must nonetheless be weighed against several limitations that define the work as hypothesis-generating. The sample was small, particularly the RRMS group (n = 7), and the discrimination confidence intervals were correspondingly wide, so the ROC analyses should be read as exploratory ranking rather than as validated classification. The leading candidate, miR-223-5p, was quantified near the detection limit, which both inflates measurement variance and mandates confirmation with a more sensitive assay. Haemolysis was not experimentally assessed in this study, and expression was normalised to a single erythrocyte-associated reference, miR-16-5p, which is sensitive to haemolysis. A post-hoc comparison showed the reference to be comparable between HC and SPMS (median raw Cq 30.1; *p* = 0.88) but modestly more abundant in the small RRMS group (median 27.9, Kruskal–Wallis *p* = 0.004), so reference-gene variability cannot be excluded as a source of bias, particularly for the low-abundance target miR-223-5p. Importantly, the SPMS versus RRMS difference in miR-223-5p persisted in raw, un-normalised Cq values (40.3 vs. 35.2), indicating that the finding is not attributable to reference-gene variation. Nonetheless, future work should incorporate haemolysis screening together with multi-reference or reference-independent absolute quantification [53,54]. In addition, EV identity and purity were not independently confirmed by EV-protein-marker immunodetection or by quantification of non-vesicular contaminants. Because the precipitation-based isolation can co-isolate lipoproteins and protein complexes, a contribution of non-vesicular materials to the measured miRNAs signal cannot be excluded and marked based purity assessment will be required in future validation. Particle concentration was not determined, as NTA or an equivalent particle-count method was not available; quantitative particle enumeration is therefore a further limitation of the EV characterisation.

Finally, the cross-sectional design cannot capture the temporal dynamics of phenotype conversion, and patients were not stratified by DMTs, which is itself known to modulate inflamma-miR levels. Moreover, the study lacks an internal validation cohort. Treatment status was partially collinear with phenotype in this cohort, the RRMS patients were treatment naïve whereas the SPMS group was, by virtue of the progressive phenotype, older and more disabled, so age, disability and treatment could not be fully separated from phenotype. These potential confounders were therefore addressed qualitatively, and confirmation will require a larger cohort in which age, disability, disease duration and treatment can be matched or adjusted for. While this cannot be fully resolved here, the direction and progressive-association of EV-miR-223-5p reproduce those reported in independent circulating datasets, providing a measure of corroboration, and we identify a prospectively recruited, adequately powered validation cohort with absolute quantification as the essential next step. We now additionally report the raw Cq characteristics of EV-miR-223-5p (median raw Cq 37.1; median inter-replicate SD 0.71 Cq; 33 of 36 samples above the reliable-detection threshold of 35), confirming that this target amplified consistently late and near the analytical detection limit of the assay.

## 4. Materials and Methods

### 4.1. Study Design and Participants

This prospective observational study was carried out at the First Neurology Clinic of the County Emergency Clinical Hospital in Târgu Mureș, Romania. Thirty-six individuals were enrolled in total: 16 patients with SPMS, 13 age- and sex-matched healthy controls (HCs), and 7 patients with RRMS, the latter included as a separate group to allow exploratory comparison across phenotypes. Because this was an exploratory discovery cohort, no prior sample-size or power calculation was performed. The RRMS group in particular reflects the limited number of eligible relapsing patients available during the enrolment window, and all downstream discrimination metrics are consequently reported with confidence intervals to make the associated statistical uncertainty explicit. For each participant, clinical information and blood samples were gathered for analysis. Patients were excluded if they had received systemic corticosteroids during the 30 days preceding enrolment, had an ongoing acute infection, or had any prior diagnosis of another autoimmune disorder.

Treatment status was recorded for all participants. The RRMS patients were treatment-naïve at sampling. The SPMS patients were sampled at baseline, prior to initiating Siponimode. Documented prior DMTs comprised in Natalizumab (n = 4), Interferon-β (n = 3) and Teriflunomide (n = 1), with no prior DMT documented in the remain patients.

Ethical approval was granted by the Ethics Committee for Scientific Research of the “George Emil Palade” University of Medicine, Pharmacy, Science and Technology of Târgu Mureș (Approval No. 2353/26 May 2023) and by the Ethics Committee of the County Emergency Clinical Hospital of Târgu Mureș (Approval No. Ad. 7749/05 April 2023). All procedures adhered to the principles set out in the Declaration of Helsinki. Before enrolment, written informed consent was secured from every patient or, where applicable, from their legally authorized representative.

### 4.2. Blood Sampling and Plasma Processing

For each participant, 20 mL of peripheral venous blood was drawn into K2-EDTA vacuum tubes (BD Vacutainer 10 mL; Becton Dickinson, Franklin Lakes, NJ, USA). Processing for plasma intended for subsequent EV isolation began within two hours of collection and involved sequential centrifugation. The samples were first spun at 300× *g* for 10 min at 4 °C, and a second spin at 2000× *g* for 20 min at 4 °C was then applied to remove any residual cellular debris. The resulting plasma supernatant was stored at −80 °C until further processing.

### 4.3. EVs Isolation and Characterization

EVs were isolated and characterized informed by the Minimal Information for Studies of Extracellular Vesicles 2023 (MISEV2023) guidelines recommendations. EVs were isolated from plasma with the ExoQuick^®^ ULTRA EV Isolation Kit for Serum and Plasma (Catalog No. EQULTRA-20A-1; System Biosciences, Palo Alto, CA, USA), which incorporates a silica-column purification step following precipitation, reducing co-isolated soluble protein and PEG carryover relative to standard precipitation protocols. The isolation followed the manufacturer’s protocol, using pre-clarified plasma samples [55]. Isolation method was selected to prioritize EV yield sufficient for downstream miRNA extraction, a trade-off consistent with MISEV2023 guidance that method choice should be matched to the intended downstream application. While precipitation-based kits show comparatively lower relative purity than size-exclusion chromatography, they consistently achieve higher EV yield and recovery, a trade-off justified when nucleic acid input, rather than proteomic-grade purity, is the primary downstream requirement.

**Figure 7:**
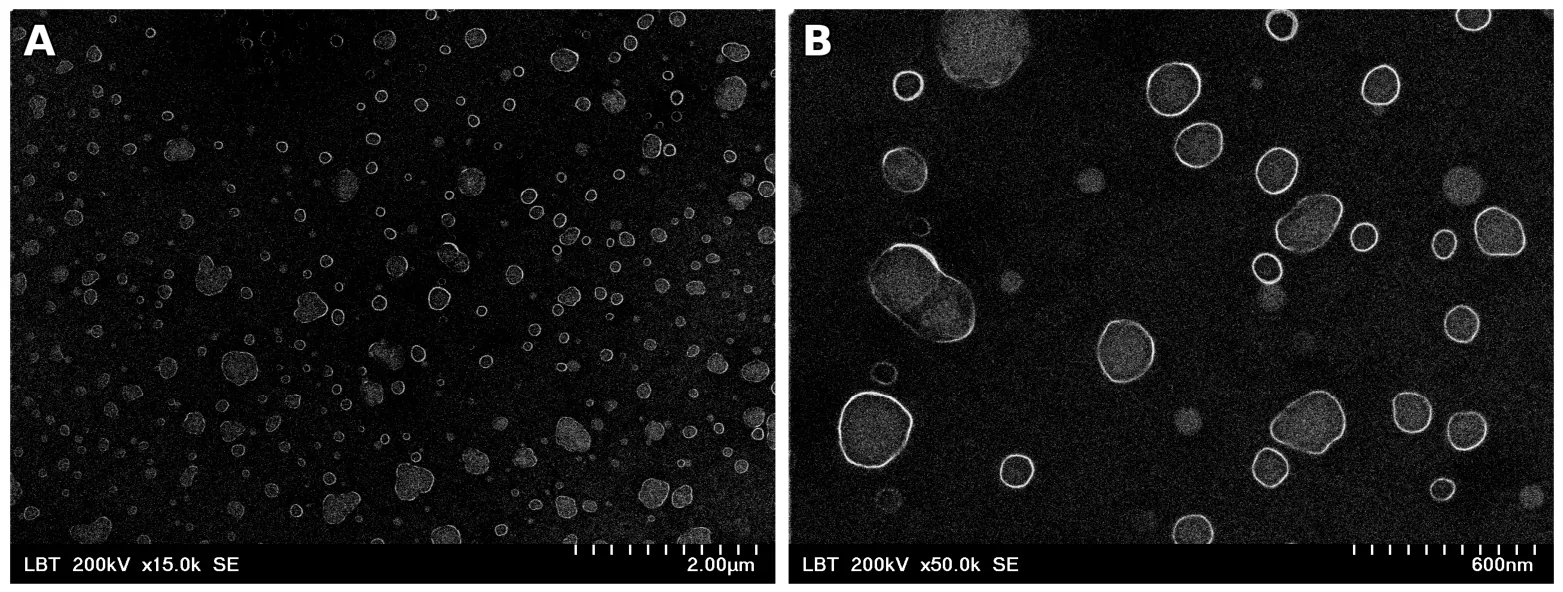
Scanning electron micrographs of plasma-derived extracellular vesicles. (**A**) Overview at ×15,000 magnification (scale bar, 2 µm) and (**B**) detail at ×50,000 (scale bar, 600 nm), showing rounded, membrane-bound vesicles consistent in size and morphology with small EVs.

EV morphology and size distribution were evaluated using a Hitachi HD-2700 scanning transmission electron microscope (STEM; Hitachi High-Tech Science Corporation, Tokyo, Japan) operated at 200 kV and magnifications of 15,000× and 50,000×. STEM imaging provided direct single-particle visualization and revealed the expected morphological heterogeneity of the EV population (Figure 7A,B).

To further characterize the physicochemical properties of the isolated EVs, measurements were performed using a Zetasizer Nano ZS90 (Malvern Instruments Ltd., Malvern, UK). Dynamic light scattering (DLS) was employed to determine the hydrodynamic diameter and polydispersity index (PDI), whereas electrophoretic light scattering was used to assess the zeta potential, an indicator of surface charge and colloidal stability. For each sample, three independent measurements were conducted, and the results were expressed as mean hydrodynamic diameter, PDI, and zeta potential values.

**Figure 8:**
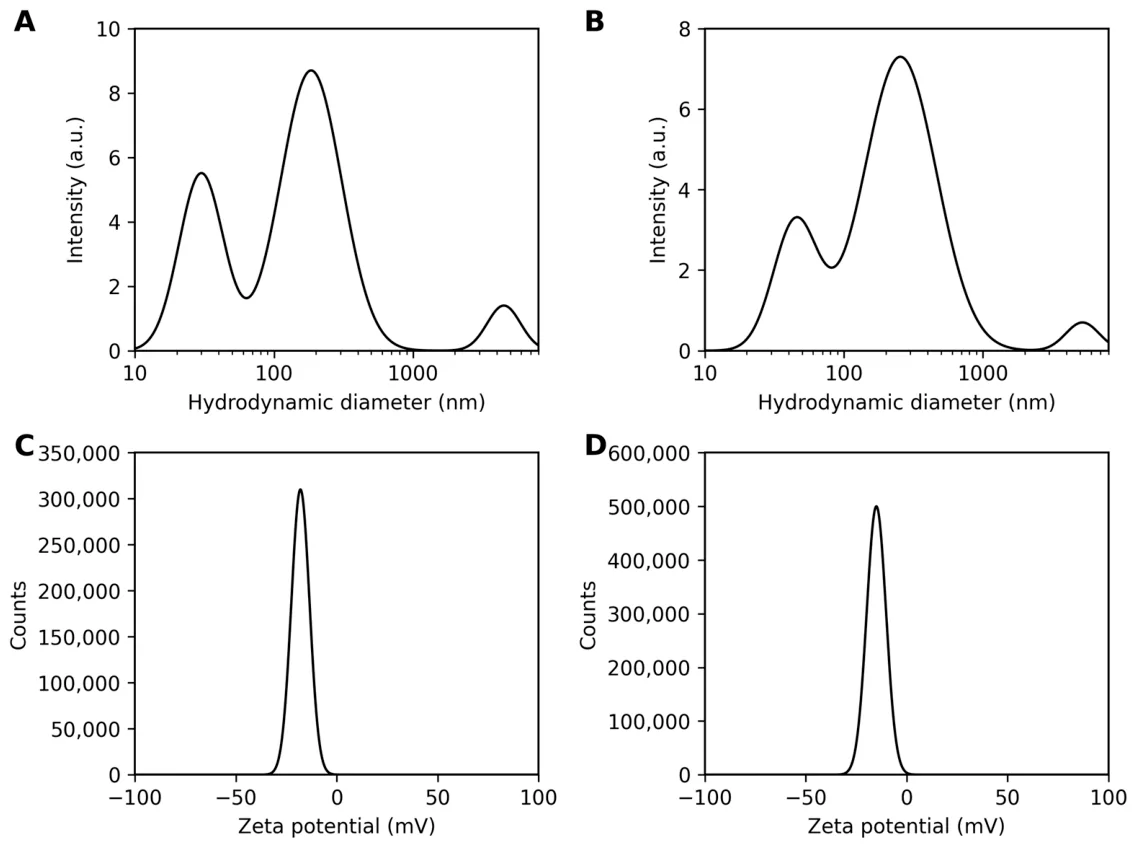
Size distribution and surface charge of plasma-derived extracellular vesicles. (**A**,**B**) Hydrodynamic diameter distributions measured by dynamic light scattering for two representative preparations. (**C**,**D**) Corresponding zeta-potential distributions, showing a net negative surface charge.

DLS analysis confirmed the heterogeneous nature of the EV preparations, revealing bimodal size distributions with peaks cantered at approximately 40–60 nm and 150–200 nm, corresponding to EV-sized and microvesicle-sized populations, respectively (Figure 8A,B). All samples exhibited PDI values greater than 0.2, consistent with the intrinsic heterogeneity of EV populations. Zeta potential measurements showed single, narrow distribution peaks with mean values ranging from −12.9 to −27.6 mV, indicating a predominantly negative surface charge characteristic of lipid membrane-bound vesicles and demonstrating sufficient colloidal stability for subsequent analyses (Figure 8C,D). EV suspensions were kept at −80 °C until analysis.

### 4.4. EV-miRNA Extraction and Expression Analysis

EV-associated miRNAs were extracted using the phenol-based mirVana™ miRNA Isolation Kit (Thermo Fisher Scientific, Waltham, MA, USA) according to the manufacturer’s instructions. Complementary DNA (cDNA) was then synthesized with the TaqMan™ MicroRNA Reverse Transcription Kit (Thermo Fisher Scientific, Waltham, MA, USA), using target-specific TaqMan™ MicroRNA primers [56]. Quantitative real-time PCR (qRT-PCR) was run on the 7500 Fast Dx Real-Time PCR System (Applied Biosystems, Waltham, MA, USA). Each reaction combined TaqMan™ Fast Advanced Master Mix (Thermo Fisher Scientific, Waltham, MA, USA) [57] with an individual TaqMan™ MicroRNA Assay directed against miR-146a-5p, miR-30a-5p, miR-155-5p, or miR-223-5p, every assay containing sequence-specific primers together with a TaqMan hydrolysis probe. Thermal cycling parameters consisted of an initial enzyme activation step at 95 °C for 20 s, followed by 40 amplification cycles of denaturation at 95 °C for 3 s and combined primer annealing and extension at 60 °C for 30 s. The endogenous small RNA miR-16-5p was used as the internal reference for normalization. Each sample was assayed in technical triplicate.

Quantification relied on the delta cycle threshold (ΔCq) approach. For each target, the cycle threshold (Cq), the number of amplification cycles needed for the fluorescent signal to rise above background, was recorded. Normalized values were then obtained by subtracting the Cq of the reference gene miR-16-5p from the Cq of each target microRNA, producing the respective delta cycle quantification (ΔCq) values.

### 4.5. Statistical Analysis

Statistical analyses were performed in Python 3.14 [58]. All comparisons were performed on subject-level ΔCt values (target Cq minus miR-16-5p Cq); fold changes (2^ΔΔCt^) are reported for biological interpretation only. Distributional assumptions were examined with the Shapiro–Wilk test (normality) and Levene’s test (homogeneity of variance). Two-group differences were assessed with the Mann–Whitney U test, complemented by Welch’s *t*-test. Omnibus differences across the three groups were assessed with the Kruskal–Wallis test and the one-way ANOVA. Effect sizes are reported as the rank-biserial correlation (derived from the Mann–Whitney U statistic) and Hedges’ g. Discrimination of SPMS from RRMS was evaluated by ROC analysis on subject-level ΔCq, with the AUC reported for each miRNA. The 95% confidence intervals for the AUC, and for ΔΔCq and the corresponding fold-changes, were obtained by non-parametric percentile bootstrap with 2000 resamples. Multiple testing was controlled with the Benjamini–Hochberg false-discovery-rate procedure: the omnibus tests were corrected as one family across the four miRNA targets, and within each miRNA, the three pairwise group comparison (RRMS vs. HC; SPMS vs. HC; SPMS vs. RRMS) were corrected as a separate family. Significance was defined as q < 0.05. Given the small groups, all inferential results, and the ROC analyses in particular, are interpreted as exploratory rather than as validated classification performance.

## 5. Conclusions

In conclusion, this study should be regarded as exploratory and hypothesis-generating rather than confirmatory, and its findings warrant validation in future independent cohorts. Within the limits of a small, cross-sectional cohort, it indicates that plasma EV-miR-223-5p may be selectively reduced in secondary-progressive disease and could help distinguish it from the relapsing–remitting form, while EV-miR-155-5p appears to mark the presence of multiple sclerosis without separating the two phenotypes; miR-30a-5p and miR-146a-5p were not informative here. These observations are best read as preliminary signals that generate testable hypotheses for future work, not as established biomarkers. Confirmation will require larger, longitudinal cohorts using sensitive absolute quantification such as ddPCR, and stratification by treatment, before any of these miRNAs could be considered for clinical use.

## Figures and Tables

**Table 1:** Raw Cq distributions of the four EV-miRNAs targets and the miR-16-5p reference across clinical groups.

miRNA	Group	n	Median Raw Cq (IQR)	Detected (% ≤ Cq 35)	Median Replicate SD (Cq)
**miR-30a-5p**	HC	13	34.3 (33.8–35.1)	69%	0.20
miR-30a-5p	RRMS	7	32.8 (32.4–33.1)	100%	0.13
miR-30a-5p	SPMS	16	34.4 (33.9–35.2)	69%	0.33
**miR-146a-5p**	HC	13	33.1 (32.7–34.7)	92%	0.18
miR-146a-5p	RRMS	7	31.8 (29.4–32.2)	100%	0.18
miR-146a-5p	SPMS	16	34.1 (32.5–35.0)	75%	0.30
**miR-155-5p**	HC	13	34.5 (33.9–34.9)	85%	0.36
miR-155-5p	RRMS	7	33.5 (32.9–33.7)	100%	0.32
miR-155-5p	SPMS	16	35.8 (35.3–36.6)	6%	0.42
**miR-223-5p**	HC	13	37.1 (36.1–38.4)	0%	0.79
miR-223-5p	RRMS	7	35.2 (34.9–36.0)	29%	0.47
miR-223-5p	SPMS	16	40.3 (37.0–41.0)	6%	0.61
**miR-16-5p (ref.)**	HC	13	30.1 (29.6–30.3)	100%	0.14
miR-16-5p (ref.)	RRMS	7	27.9 (26.8–28.3)	100%	0.07
miR-16-5p (ref.)	SPMS	16	30.1 (28.9–30.7)	100%	0.13

Values are per-sample raw Cq (Mean Equivalent Cq) from RT-qPCR; median (interquartile range). “Detected” is the percentage of samples amplifying at or below Cq 35, the reliable-detection threshold. Median replicate SD is the within-sample dispersion across technical replicates. HC, healthy controls (n = 13); RRMS (n = 7); SPMS (n = 16, baseline). miR-16-5p is the endogenous normalizer.

**Table 2:** Group-level exosomal miRNA expression and fold-change relative to healthy controls (ΔCt normalized to miR-16-5p; n = 13 HC, 7 RRMS, 16 SPMS).

miRNA	HC ΔCq	RRMS ΔCq	SPMS ΔCq	FC RRMS	FC SPMS	Trend
miR-30a-5p	3.97	4.97	4.63	0.48	0.74	↓
**miR-223-5p**	**7.34**	**7.79**	**9.05**	**0.54**	**0.24**	**↓↓**
**miR-155-5p**	**4.31**	**5.62**	**5.73**	**0.28**	**0.32**	**↓**
miR-146a-5p	3.46	3.51	3.95	1.01	0.72	–

ΔCq values are group medians; FC columns are the fold change of each disease group versus HC.

**Table 3:** Inferential tests: omnibus Kruskal–Wallis across the three groups and pairwise Mann–Whitney U tests on ΔCq (FDR = Benjamini–Hochberg correction within each miRNA).

miRNA	KW FDR	RRMS vs. HC	SPMS vs. HC	SPMS vs. RRMS
miR-30a-5p	0.18 (ns)	*p* = 0.115	*p* = 0.283	*p* = 0.222
**miR-223-5p**	**0.024**	***p* = 0.351 (ns)**	***p* = 0.005 (FDR 0.021)**	***p* = 0.089 (trend)**
**miR-155-5p**	**0.024**	***p* = 0.014 (FDR 0.019)**	***p* = 0.006 (FDR 0.012)**	***p* = 0.922 (ns)**
miR-146a-5p	0.18 (ns)	*p* = 0.817	*p* = 0.083	*p* = 0.222

Only miR-223-5p and miR-155-5p reached significance in the omnibus test (FDR = 0.024). ns = not significant.

**Table 4:** Direct RRMS vs. SPMS comparison with effect size and discrimination metrics (subject-level ΔCt). FC = expression ratio SPMS/RRMS; r_rb_ = rank-biserial effect size; and AUC = discrimination of SPMS from RRMS (bootstrap 95% CI).

miRNA	RRMS ΔCq	SPMS ΔCq	FC SPMS/RRMS	MWU *p*	r_rb_	AUC
**miR-223-5p**	**7.79**	**9.05**	**0.44 ↓**	**0.089**	**−0.46**	**0.73**
miR-30a-5p	4.97	4.63	1.53 ↑	0.222	+0.34	0.67
miR-146a-5p	3.51	3.95	0.72 ↓	0.222	−0.34	0.67
miR-155-5p	5.62	5.73	1.16 ≈	0.922	+0.04	0.52

Rows are ordered by discrimination ability. FC > 1 indicates higher expression in SPMS, FC < 1 indicates lower expression; the arrow marks direction. Only miR-223 (highlighted) separated the phenotypes above chance.

## Data Availability

The datasets generated and analysed during the current study are available from the corresponding author upon reasonable request.

## Abbreviations

The following abbreviations are used in this manuscript:

AUC	area under the curve
BBB	blood–brain barrier
CD4^+^	cluster of differentiation
cDNA	complementary DNA
CI	confidence interval
CNS	central nervous system
Cq	quantification cycle
CSF	cerebrospinal fluid
ΔCq	delta quantification cycle
ΔΔCt	comparative threshold cycle
ddPCR	droplet digital PCR
DLS	dynamic light scattering
DMTs	disease-modifying therapies
EAE	experimental autoimmune encephalomyelitis
EV	extracellular vesicle
EV-miRNAs	extracellular vesicle-associated microRNAs
FC	fold change
FDR	false discovery rate
HC	healthy controls
KW	Kruskal–Wallis
miRNA	microRNA
MISEV2023	Minimal Information for Studies of Extracellular Vesicles 2023
mRNA	messenger RNA
MRI	magnetic resonance imaging
MS	multiple sclerosis
MWU	Mann–Whitney U test
NF-κB	nuclear factor kappa B
NS	not significant
PBMCs	peripheral blood mononuclear cells
PDI	polydispersity index
PIRA	progression independent of relapse activity
PPMS	primary progressive multiple sclerosis
RRMS	relapsing–remitting multiple sclerosis
qRT-PCR	quantitative reverse-transcription PCR
ROC	receiver operating characteristic
RQ	relative quantification
RRB	rank-biserial correlation
SPMS	secondary-progressive disease
STEM	scanning transmission microscopy
Th1/Th1	T-helper 1/17

## References

[1] Bose G., Thebault S.D.X., Fadda G., Brooks J.A., Freedman M.S. (2025). Role of soluble biomarkers in treating multiple sclerosis and neuroinflammatory conditions. Neurotherapeutics.

[2] Kapoor R., Smith K.E., Allegretta M., Arnold D.L., Carroll W., Comabella M., Furlan R., Harp C., Kuhle J., Leppert D. (2020). Serum neurofilament light as a biomarker in progressive multiple sclerosis. Neurology.

[3] Konitsioti A.M., Schweitzer F., Johannis W., Steffen F., Bittner S., Fink G.R., Schroeter M., Warnke C. (2025). Serum neurofilament light chain as a biomarker of disease control in multiple sclerosis: A real-world cross-sectional analysis of therapeutic regimens. J. Neurol..

[4] Di Filippo M., Gaetani L., Centonze D., Hegen H., Kuhle J., Teunissen C.E., Tintoré M., Villar L.M., Willemse E.A.J., Zetterberg H. (2024). Fluid biomarkers in multiple sclerosis: From current to future applications. Lancet Reg. Health Eur..

[5] Chitnis T., Prat A. (2020). A roadmap to precision medicine for multiple sclerosis. Mult. Scler..

[6] Portaccio E., Magyari M., Havrdova E.K., Ruet A., Brochet B., Scalfari A., Di Filippo M., Tur C., Montalban X., Amato M.P. (2024). Multiple sclerosis: Emerging epidemiological trends and redefining the clinical course. Lancet Reg. Health Eur..

[7] Fuh-Ngwa V., Charlesworth J.C., Zhou Y., van der Mei I., Melton P.E., Broadley S.A., Ponsonby A.L., Simpson-Yap S., Lechner-Scott J., Taylor B.V. (2023). The association between disability progression, relapses, and treatment in early relapse onset MS: An observational, multi-centre, longitudinal cohort study. Sci. Rep..

[8] Bastos A., Soares M., Guimarães J. (2024). Markers of secondary progression in multiple sclerosis. Mult. Scler. Relat. Disord..

[9] Cree B.A.C., Arnold D.L., Chataway J., Chitnis T., Fox R.J., Pozo Ramajo A., Murphy N., Lassmann H. (2021). Secondary Progressive Multiple Sclerosis: New Insights. Neurology.

[10] Scalfari A., Neuhaus A., Daumer M., Muraro P.A., Ebers G.C. (2014). Onset of secondary progressive phase and long-term evolution of multiple sclerosis. J. Neurol. Neurosurg. Psychiatry.

[11] Oh J., Alikhani K., Bruno T., Devonshire V., Giacomini P.S., Giuliani F., Nakhaipour H.R., Schecter R., Larochelle C. (2019). Diagnosis and management of secondary-progressive multiple sclerosis: Time for change. Neurodegener. Dis. Manag..

[12] Katz Sand I., Krieger S., Farrell C., Miller A.E. (2014). Diagnostic uncertainty during the transition to secondary progressive multiple sclerosis. Mult. Scler..

[13] Maier S., Barcutean L., Andone S., Manu D., Sarmasan E., Bajko Z., Balasa R. (2023). Recent Progress in the Identification of Early Transition Biomarkers from Relapsing-Remitting to Progressive Multiple Sclerosis. Int. J. Mol. Sci..

[14] Dickens A.M., Larkin J.R., Griffin J.L., Cavey A., Matthews L., Turner M.R., Wilcock G.K., Davis B.G., Claridge T.D., Palace J. (2014). A type 2 biomarker separates relapsing-remitting from secondary progressive multiple sclerosis. Neurology.

[15] Torres-Iglesias G., López-Molina M., Ayala-Suárez R., Egido B., Laso-García F., Chamorro B., Puertas I., Fernández-Fournier M., Montero-Calle A., Barderas R. (2025). Extracellular Vesicle-Derived MicroRNAs as a Biomarker for Therapeutic Response in Multiple Sclerosis. Neurol. Neuroimmunol. Neuroinflammation.

[16] Emami Nejad A., Mostafavi Zadeh S.M., Nickho H., Sadoogh Abbasian A., Forouzan A., Ahmadlou M., Nedaeinia R., Shaverdi S., Manian M. (2023). The role of microRNAs involved in the disorder of blood-brain barrier in the pathogenesis of multiple sclerosis. Front. Immunol..

[17] Geraci F., Ragonese P., Barreca M.M., Aliotta E., Mazzola M.A., Realmuto S., Vazzoler G., Savettieri G., Sconzo G., Salemi G. (2018). Differences in Intercellular Communication During Clinical Relapse and Gadolinium-Enhanced MRI in Patients With Relapsing Remitting Multiple Sclerosis: A Study of the Composition of Extracellular Vesicles in Cerebrospinal Fluid. Front. Cell. Neurosci..

[18] Manna I., De Benedittis S., Porro D. (2024). Extracellular Vesicles in Multiple Sclerosis: Their Significance in the Development and Possible Applications as Therapeutic Agents and Biomarkers. Genes.

[19] Sáenz-Cuesta M., Osorio-Querejeta I., Otaegui D. (2014). Extracellular Vesicles in Multiple Sclerosis: What are They Telling Us?. Front. Cell. Neurosci..

[20] Liu Q., Siadat S.D. (2026). MicroRNA-155 in neurodegeneration. Clin. Chim. Acta.

[21] O’Brien J., Hayder H., Zayed Y., Peng C. (2018). Overview of MicroRNA Biogenesis, Mechanisms of Actions, and Circulation. Front. Endocrinol..

[22] Juźwik C.A., Drake S.S., Zhang Y., Paradis-Isler N., Sylvester A., Amar-Zifkin A., Douglas C., Morquette B., Moore C.S., Fournier A.E. (2019). microRNA dysregulation in neurodegenerative diseases: A systematic review. Prog. Neurobiol..

[23] McCoy C.E. (2017). miR-155 Dysregulation and Therapeutic Intervention in Multiple Sclerosis. Adv. Exp. Med. Biol..

[24] Vistbakka J., Elovaara I., Lehtimäki T., Hagman S. (2017). Circulating microRNAs as biomarkers in progressive multiple sclerosis. Mult. Scler..

[25] Ahmed Ali M., Gamil Shaker O., Mohamed Eid H., Elsayed Mahmoud E., Mahmoud Ezzat E., Nady Gaber S. (2020). Relationship between miR-155 and miR-146a polymorphisms and susceptibility to multiple sclerosis in an Egyptian cohort. Biomed. Rep..

[26] Maciak K., Dziedzic A., Miller E., Saluk-Bijak J. (2021). miR-155 as an Important Regulator of Multiple Sclerosis Pathogenesis. A Review. Int. J. Mol. Sci..

[27] Pietrasik S., Dziedzic A., Miller E., Starosta M., Saluk-Bijak J. (2021). Circulating miRNAs as Potential Biomarkers Distinguishing Relapsing–Remitting from Secondary Progressive Multiple Sclerosis. A Review. Int. J. Mol. Sci..

[28] Wang H. (2021). MicroRNAs, Multiple Sclerosis, and Depression. Int. J. Mol. Sci..

[29] Martinez B., Peplow P.V. (2020). MicroRNAs in blood and cerebrospinal fluid as diagnostic biomarkers of multiple sclerosis and to monitor disease progression. Neural Regen. Res..

[30] Fan Y., Huang H., Shao J., Huang W. (2023). MicroRNA-mediated regulation of reactive astrocytes in central nervous system diseases. Front. Mol. Neurosci..

[31] Mosora O., Maier S., Manu D., Bărcuțean L., Roman M., Dumitreasă M., Bălașa R. (2025). Exosomal microRNAs as Early Transition Biomarkers from Recurrent-Remissive to Secondary Progressive Multiple Sclerosis. Int. J. Mol. Sci..

[32] Ebrahimkhani S., Vafaee F., Young P.E., Hur S.S.J., Hawke S., Devenney E., Beadnall H., Barnett M.H., Suter C.M., Buckland M.E. (2017). Exosomal microRNA signatures in multiple sclerosis reflect disease status. Sci. Rep..

[33] Wang K., Yuan Y., Cho J.H., McClarty S., Baxter D., Galas D.J. (2012). Comparing the MicroRNA spectrum between serum and plasma. PLoS ONE.

[34] Max K.E.A., Bertram K., Akat K.M., Bogardus K.A., Li J., Morozov P., Ben-Dov I.Z., Li X., Weiss Z.R., Azizian A. (2018). Human plasma and serum extracellular small RNA reference profiles and their clinical utility. Proc. Natl. Acad. Sci. USA.

[35] Mohammadinasr M., Montazersaheb S., Ayromlou H., Hosseini V., Molavi O., Hejazi M.S. (2024). Exosome Content-Mediated Signaling Pathways in Multiple Sclerosis. Mol. Neurobiol..

[36] Junker A., Krumbholz M., Eisele S., Mohan H., Augstein F., Bittner R., Lassmann H., Wekerle H., Hohlfeld R., Meinl E. (2009). MicroRNA profiling of multiple sclerosis lesions identifies modulators of the regulatory protein CD47. Brain.

[37] Ridolfi E., Fenoglio C., Cantoni C., Calvi A., De Riz M., Pietroboni A., Villa C., Serpente M., Bonsi R., Vercellino M. (2013). Expression and Genetic Analysis of MicroRNAs Involved in Multiple Sclerosis. Int. J. Mol. Sci..

[38] Hosseini A., Ghaedi K., Tanhaei S., Ganjalikhani-Hakemi M., Teimuri S., Etemadifar M., Nasr Esfahani M.H. (2016). Upregulation of CD4+T-Cell Derived MiR-223 in The Relapsing Phase of Multiple Sclerosis Patients. Cell J..

[39] Cantoni C., Cignarella F., Ghezzi L., Mikesell B., Bollman B., Berrien-Elliott M.M., Ireland A.R., Fehniger T.A., Wu G.F., Piccio L. (2017). Mir-223 regulates the number and function of myeloid-derived suppressor cells in multiple sclerosis and experimental autoimmune encephalomyelitis. Acta Neuropathol..

[40] Fenoglio C., Ridolfi E., Cantoni C., De Riz M., Bonsi R., Serpente M., Villa C., Pietroboni A.M., Naismith R.T., Alvarez E. (2013). Decreased circulating miRNA levels in patients with primary progressive multiple sclerosis. Mult. Scler..

[41] Vistbakka J., Sumelahti M.L., Lehtimäki T., Hagman S. (2022). Temporal variability of serum miR-191, miR-223, miR-128, and miR-24 in multiple sclerosis: A 4-year follow-up study. J. Neurol. Sci..

[42] Lopez-Ramirez M.A., Wu D., Pryce G., Simpson J.E., Reijerkerk A., King-Robson J., Kay O., de Vries H.E., Hirst M.C., Sharrack B. (2014). MicroRNA-155 negatively affects blood-brain barrier function during neuroinflammation. FASEB J..

[43] Moore C.S., Rao V.T., Durafourt B.A., Bedell B.J., Ludwin S.K., Bar-Or A., Antel J.P. (2013). miR-155 as a multiple sclerosis-relevant regulator of myeloid cell polarization. Ann. Neurol..

[44] Paraboschi E.M., Soldà G., Gemmati D., Orioli E., Zeri G., Benedetti M.D., Salviati A., Barizzone N., Leone M., Duga S. (2011). Genetic association and altered gene expression of mir-155 in multiple sclerosis patients. Int. J. Mol. Sci..

[45] Zhang J., Cheng Y., Cui W., Li M., Li B., Guo L. (2014). MicroRNA-155 modulates Th1 and Th17 cell differentiation and is associated with multiple sclerosis and experimental autoimmune encephalomyelitis. J. Neuroimmunol..

[46] Murugaiyan G., Beynon V., Mittal A., Joller N., Weiner H.L. (2011). Silencing microRNA-155 ameliorates experimental autoimmune encephalomyelitis. J. Immunol..

[47] Devier D.J., Lovera J.F., Lukiw W.J. (2015). Increase in NF-κB-sensitive miRNA-146a and miRNA-155 in multiple sclerosis (MS) and pro-inflammatory neurodegeneration. Front. Mol. Neurosci..

[48] Giuliani A., Lattanzi S., Ramini D., Graciotti L., Danni M.C., Procopio A.D., Silvestrini M., Olivieri F., Sabbatinelli J. (2021). Potential prognostic value of circulating inflamma-miR-146a-5p and miR-125a-5p in relapsing-remitting multiple sclerosis. Mult. Scler. Relat. Disord..

[49] Muñoz-San Martín M., Reverter G., Robles-Cedeño R., Buxò M., Ortega F.J., Gómez I., Tomàs-Roig J., Celarain N., Villar L.M., Perkal H. (2019). Analysis of miRNA signatures in CSF identifies upregulation of miR-21 and miR-146a/b in patients with multiple sclerosis and active lesions. J. Neuroinflammation.

[50] Manna I., Iaccino E., Dattilo V., Barone S., Vecchio E., Mimmi S., Filippelli E., Demonte G., Polidoro S., Granata A. (2018). Exosome-associated miRNA profile as a prognostic tool for therapy response monitoring in multiple sclerosis patients. FASEB J..

[51] Shademan B., Nourazarian A., Nikanfar M., Biray Avci C., Hasanpour M., Isazadeh A. (2020). Investigation of the miRNA146a and miRNA155 gene expression levels in patients with multiple sclerosis. J. Clin. Neurosci..

[52] Groen K., Maltby V.E., Lea R.A., Sanders K.A., Fink J.L., Scott R.J., Tajouri L., Lechner-Scott J. (2018). Erythrocyte microRNA sequencing reveals differential expression in relapsing-remitting multiple sclerosis. BMC Med. Genom..

[53] Kirschner M.B., Kao S.C., Edelman J.J., Armstrong N.J., Vallely M.P., van Zandwijk N., Reid G. (2011). Haemolysis during sample preparation alters microRNA content of plasma. PLoS ONE.

[54] Donati S., Ciuffi S., Brandi M.L. (2019). Human Circulating miRNAs Real-time qRT-PCR-based Analysis: An Overview of Endogenous Reference Genes Used for Data Normalization. Int. J. Mol. Sci..

[55] ExoQuick^®^ ULTRA EV Isolation System. https://www.systembio.com/products/exosome-research/exosome-isolation/exoquick-ultra/serum-and-plasma-0/the-purest-and-highest-yielding-ev-isolation-system.

[56] https://documents.thermofisher.com/TFS-Assets/LSG/manuals/cms_055423.pdf.

[57] https://documents.thermofisher.com/TFS-Assets/LSG/manuals/4364031_TaqSmallRNA_UG.pdf.

[58] Python Software Foundation (2025). Python Language Reference, *version 3.14*.

